# Molecularly specific detection of bacterial lipoteichoic acid for diagnosis of prosthetic joint infection of the bone

**DOI:** 10.1038/s41413-018-0014-y

**Published:** 2018-04-25

**Authors:** Julie E. Pickett, John M. Thompson, Agnieszka Sadowska, Christine Tkaczyk, Bret R. Sellman, Andrea Minola, Davide Corti, Antonio Lanzavecchia, Lloyd S. Miller, Daniel LJ Thorek

**Affiliations:** 1 Division of Nuclear Medicine and Molecular Imaging, Department of Radiology and Radiological Science, Johns Hopkins University School of Medicine, Gaithersburg, MD USA; 2 Department of Orthopaedic Surgery, Johns Hopkins University School of Medicine, Baltimore, MD 21205 USA; 3 Department of Infectious Disease, MedImmune LLC, Gaithersburg, MD USA; 4 Humabs BioMed SA, Bellinzona, Switzerland; 5 Institute for Research in Biomedicine, Università della Svizzera italiana, Bellinzona, Switzerland; 6 Department of Dermatology, Johns Hopkins University School of Medicine, Baltimore, MD 21205 USA; 7 Division of Infectious Disease, Department of Medicine, Johns Hopkins University School of Medicine, Baltimore, MD USA; 8 Cancer Molecular and Functional Imaging Program, Department of Oncology, Sidney Kimmel Comprehensive Cancer Center, Johns Hopkins University School of Medicine, Baltimore, MD 21205 USA

## Abstract

Discriminating sterile inflammation from infection, especially in cases of aseptic loosening versus an actual prosthetic joint infection, is challenging and has significant treatment implications. Our goal was to evaluate a novel human monoclonal antibody (mAb) probe directed against the Gram-positive bacterial surface molecule lipoteichoic acid (LTA). Specificity and affinity were assessed in vitro. We then radiolabeled the anti-LTA mAb and evaluated its effectiveness as a diagnostic imaging tool for detecting infection via immunoPET imaging in an in vivo mouse model of prosthetic joint infection (PJI). In vitro and ex vivo binding of the anti-LTA mAb to pathogenic bacteria was measured with Octet, ELISA, and flow cytometry. The in vivo PJI mouse model was assessed using traditional imaging modalities, including positron emission tomography (PET) with [^18^F]FDG and [^18^F]NaF as well as X-ray computed tomography (CT), before being evaluated with the zirconium-89-labeled antibody specific for LTA ([^89^Zr]SAC55). The anti-LTA mAb exhibited specific binding in vitro to LTA-expressing bacteria. Results from imaging showed that our model could reliably simulate infection at the surgical site by bioluminescent imaging, conventional PET tracer imaging, and bone morphological changes by CT. One day following injection of both the radiolabeled anti-LTA and isotype control antibodies, the anti-LTA antibody demonstrated significantly greater (*P* < 0.05) uptake at *S*. *aureus*-infected prosthesis sites over either the same antibody at sterile prosthesis sites or of control non-specific antibody at infected prosthesis sites. Taken together, the radiolabeled anti-LTA mAb, [^89^Zr]SAC55, may serve as a valuable diagnostic molecular imaging probe to help distinguish between sterile inflammation and infection in the setting of PJI. Future studies are needed to determine whether these findings will translate to human PJI.

## Joint replacements: Distinguishing infection from inflammation

A new imaging technique distinguishes bacterial infection at the site of joint implants from less-serious postoperative inflammation, saving patients from unnecessary and invasive treatments. Daniel Thorek of Johns Hopkins University School of Medicine and colleagues used an antibody that binds to lipoteichoic acid on the cell wall of *Staphylococcus* bacteria to detect infection at joint implant sites. The antibody was labeled with a radioactive agent and injected into mice that simulated infection of a knee replacement site. A PET scan conducted 1 day after antibody injection showed that it gathered at the infected joint significantly more than it did at the uninfected implant sites in other mice. This method could improve the diagnosis of joint implant infection, which necessitates removal of the prosthetic and all infected tissues, followed by prolonged antibiotic therapy.

## Introduction

Total joint arthroplasty is a common orthopedic surgical procedure with not only significant benefits but also challenging complications, especially related to infection.^1^ Prosthetic joint infection (PJI), which is an infection involving the implant and adjacent bone and joint tissues, affects up to 2% of all primary arthroplasties, resulting in an estimated 25 000 cases and costs exceeding $3.2 billion.^2^ The current standard of care therapy for chronic PJI of the hip and knee involves complete removal of all infected tissue and implants with eventual hardware replacement after prolonged antibiotic therapy. Successful treatment occurs in (65–90)% of cases, but failure often requires further treatments, surgeries, or even amputation.^3,4^ As the total number of total hip and total knee arthroplasties in the United States is projected to exceed 4 million by the year 2030, these complications will only become more common.^2^

Differentiating PJI from sterile inflammation in the postoperative period remains a challenge for contemporary diagnostic techniques, such as commonly used imaging modalities, laboratory tests, and bacterial cultures.^5^ Noninvasive imaging modalities are attractive options, as they offer the potential for diagnosing, monitoring, and assessing treatment longitudinally if sufficient sensitivity and resolution can be achieved. X-ray and magnetic resonance imaging (MRI) reveal gross morphological changes but unfortunately produce implant-related artifacts (especially involving metallic implants) that obscure relevant changes in the surrounding tissue.

Positron emission tomography (PET) generates three-dimensional (3D) tracer concentration images with the potential for excellent sensitivity and without implant artifacts suffered by computed tomography (CT) and MRI. The sensitivity of this modality has the ability to detect infectious burden and molecular changes that occur prior to macroscopic anatomical alterations.^6^ Widely used conventional tracers, including [^18^F]FDG (2-fluoro-2-deoxy-d-glucose), [^18^F]NaF, and [^68^Ga]citrate, have been investigated for PET localization and evaluation of PJI with variable success.^7–10^ However, current imaging approaches assess indirect markers of infection—methods that are often hindered by low specificity and/or sensitivity—rather than directly targeting the infectious agent.

An alternative approach to improve diagnosis of PJI may be to use targeted probes, such as antibodies or peptides.^11^ Radiolabeled antibodies for immunoPET have long been used for investigational and clinical studies for oncological applications.^12^ Prior infection-related investigations have focused on antibodies to human targets such as white blood cells or other indirect markers of infection.^7,8^ In this study, we assessed an antibody directly targeting Gram-positive bacteria such as *Staphylococcus aureus* and *Staphylococcus epidermidis* (the bacteria commonly responsible for PJI). The antibody is directed against lipoteichoic acid (LTA), a surface-associated component of the cell wall of Gram-positive bacteria. Expressed by the bacteria at high levels, this antigen is a promising target for directed imaging and therapeutic agents. By directly targeting the infectious agent, we demonstrate improved diagnostic capabilities of noninvasive imaging, which may guide improved patient management.

## Results

### In vivo model of a *S. aureus* PJI

**Fig. 1:**
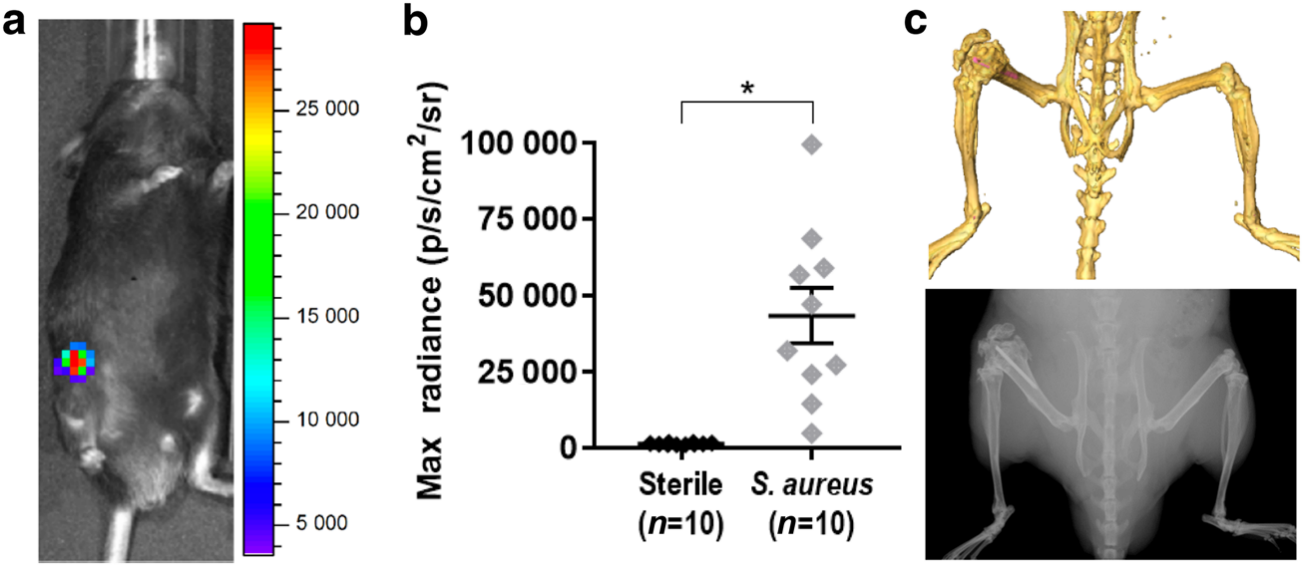
In vivo bioluminescent imaging in a mouse model of PJI. A titanium Kirschner-wire was surgically placed in the mouse femur with or without an inoculum of a bioluminescent *S*. *aureus* strain in the knee joint prior to closure. **a** Representative in vivo bioluminescent signals at the *S*. *aureus*-infected postoperative site. **b** Maximum radiance values for individuals (mean ± S.E.M.) with sterile control implants or *S*. *aureus*-infected postoperative distal femur sites at a representative time point (1 400 ± 90 vs 43 000 ± 9 000, respectively). **c** CT (top) and X-ray (bottom) imaging demonstrating the location of the Kirschner-wire (false-colored pink in the CT image) in the distal femur. *P < 0.000 6

Previous work with other bioluminescent strains has demonstrated that bioluminescent signals closely approximate and correlate with the bacterial burden, as measured by ex vivo colony-forming unit (CFU) counting.^13–17^ In vivo bioluminescence imaging (BLI) of mice demonstrated that the average signal from the *S*. *aureus*-infected postoperative legs (4.3 ± 0.9) × 10^4^ p/s/cm^2^/sr (*n* = 10) was greater than for mice that had surgery performed without bacterial inoculation (1.4 ± 0.09) × 10^3^ p/s/cm^2^/sr (*n* = 10, *P* < 0.000 6) (Fig. 1a, b). The infection remained locally at the original site of inoculation as bioluminescent signals were not detected above background at other regions of the body. In addition, this model of PJI resulted in expansion and damage of the distal femur as the infection progressed, which was confirmed by CT and high-resolution X-ray imaging (Fig. 1c).^16^ The anatomical changes in the distal femur of the *S*. *aureus*-infected postoperative legs can be seen clearly compared with the contralateral, non-operative leg.

### [^18^F]FDG imaging

**Fig. 2:**
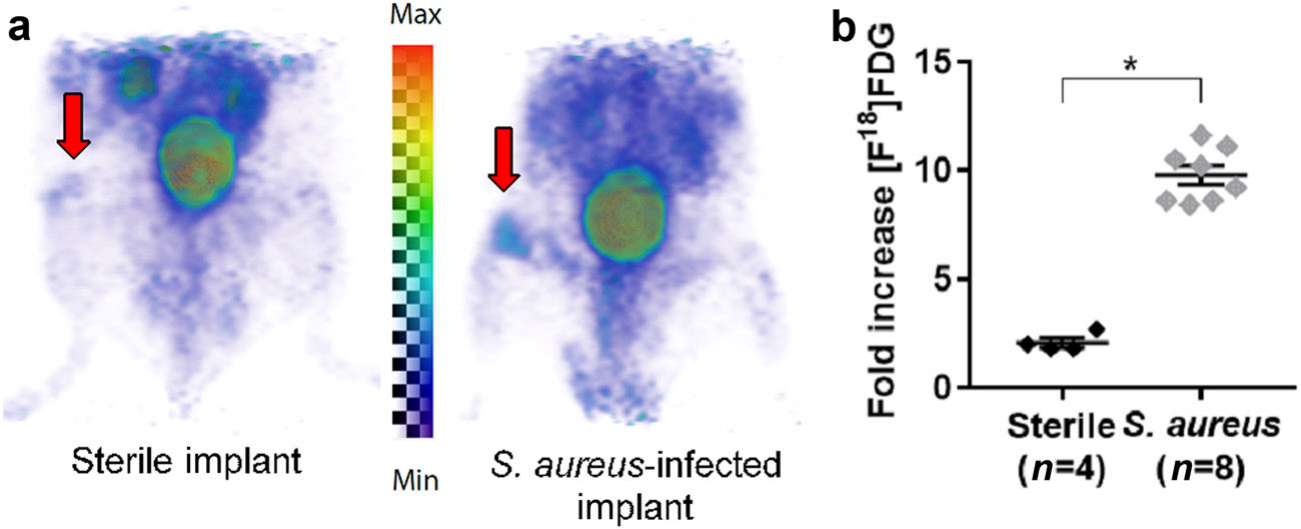
[^18^F]FDG uptake in the mouse model of PJI. **a** Representative [^18^F]FDG PET images (arrows indicate the operative site on the distal femur). **b** Mean ± S.E.M. fold increase of the [^18^F]FDG signal in the sterile vs *S*. *aureus*-infected, operative legs over the contralateral, non-operative legs (2.1 ± 0.2 vs 9.8 ± 0.4, respectively). *P < 0.000 1

[^18^F]FDG is a glucose analog that is taken up by cells with high glucose avidity, including tumors, inflammation, and bacteria. Volumes of interest were defined manually around areas of focal [^18^F]FDG uptake in the distal femurs of both the postoperative and contralateral, non-operative legs of the mice after saline injection or *S*. *aureus* infection. To control for variability in mouse size, data are presented as averages of the fold increase of the postoperative leg measurement over the contralateral, non-operative leg. In the *S*. *aureus*-infected postoperative legs, there was a large mean increase (9.8 ± 0.4, *n* = 8) compared with a doubling (2.1 ± 0.2, *n* = 4) for the sterile postoperative legs (Fig. 2). We see a significant uptake of in *S*. *aureus*-infected surgical postoperative legs compared to contralateral non-operative legs (*P* < 0.000 1), suggesting that the probe specifically localized to infected surgical sites. However, the glucose avidity of metabolically active cells, such as immune cells at sites of inflammation following sterile implantation surgery, complicate diagnosis of infection.^18^

### Bone scans with [^18^F]NaF

**Fig. 3:**
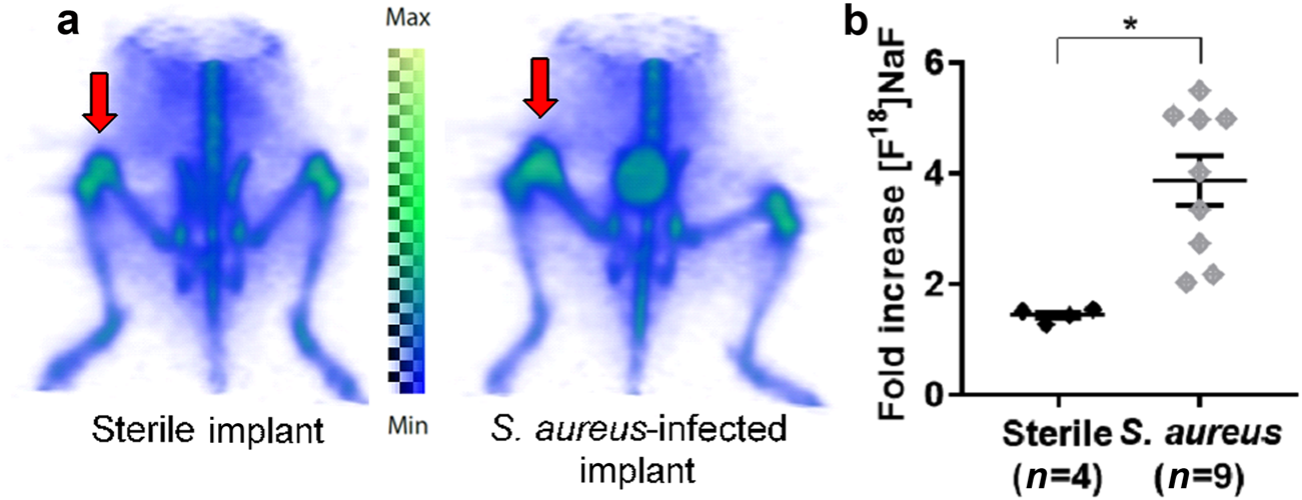
[^18^F]NaF uptake in the mouse model of PJI. **a** Representative [^18^F]NaF PET images (arrows indicate the operative site on the distal femur). **b** Mean ± S.E.M. fold increase of the [^18^F]NaF signal in the sterile vs *S*. *aureus*-infected, operative legs over the contralateral, non-operative legs (1.4 ± 0.06 vs 3.9 ± 0.4, respectively). *P < 0.000 3

[^18^F]NaF is incorporated within the bone matrix at sites of increased bone turnover such as that with natural growth at the physis as well as at sites of reactive bone changes that occur in response to infection.^19–22^ [^18^F]NaF uptake was evaluated in this PJI model, and greater [^18^F]NaF uptake was observed in the *S*. *aureus*-infected postoperative legs (3.9 ± 0.4 fold, *n* = 9) than in the sterile postoperative legs (1.4 ± 0.06 fold, *n* = 4, *P* < 0.000 3) (Fig. 3a, b). Of note, the fold increase observed in the sterile postoperative leg compared with the contralateral non-operative leg was >1.0, which was likely due to modest bone turnover from the naturally occurring inflammatory response from the implant alone.

### CT imaging

**Fig. 4:**
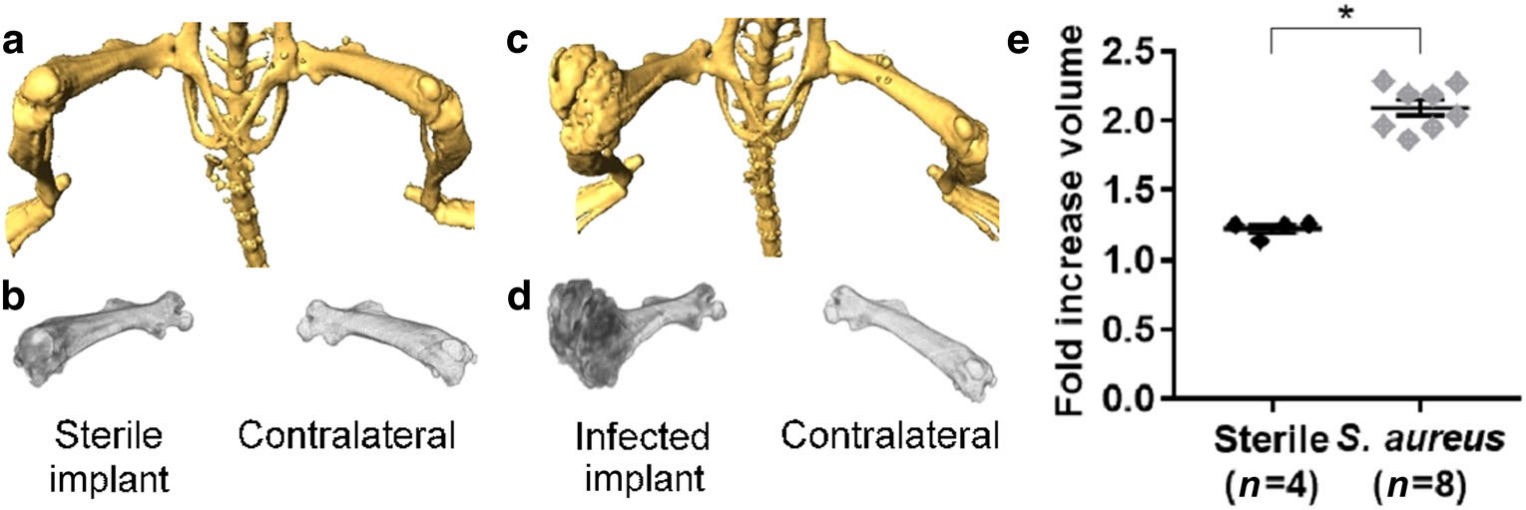
CT imaging in the mouse model of PJI. **a**–**d** CT imaging of the femur (**a**,** c**) and corresponding volumetric measurement (**b**, **d**) shown for representative sterile (**a**,** b**) and *S*. *aureus*-infected (**c**, **d**) mice. **e** Mean ± S.E.M. fold increase of the femur volumes in the sterile vs *S*. *aureus*-infected operative legs over the contralateral, non-operative legs (1.2 ± 0.03 vs 2.1 ± 0.06, respectively). *P < 0.000 1

Femoral volumes were defined from CT scan data. To control for variability in the size of the femurs between the individual mice, data are presented as averages of the fold increase of the measurement of the postoperative femur over the contralateral, non-operative femur. The femur volumes of *S*. *aureus*-infected postoperative legs had a doubling of bone volume for the entire femur (2.1 ± 0.06, *n* = 8) compared with sterile, postoperative legs, which had a slight increase over their non-operative counterpart (1.2 ± 0.03, *n* = 4, *P* < 0.000 1), the large size and structural differences of which can be seen in representative scans (Fig. 4a–e). Taken together, CT data analysis showed that the *S*. *aureus*-infected operative legs had a marked expansion of the femur volume compared with sterile postoperative legs, which is consistent with our previous observations.^16,23^

### Specificity of the anti-LTA monoclonal antibody (mAb)

**Fig. 5:**
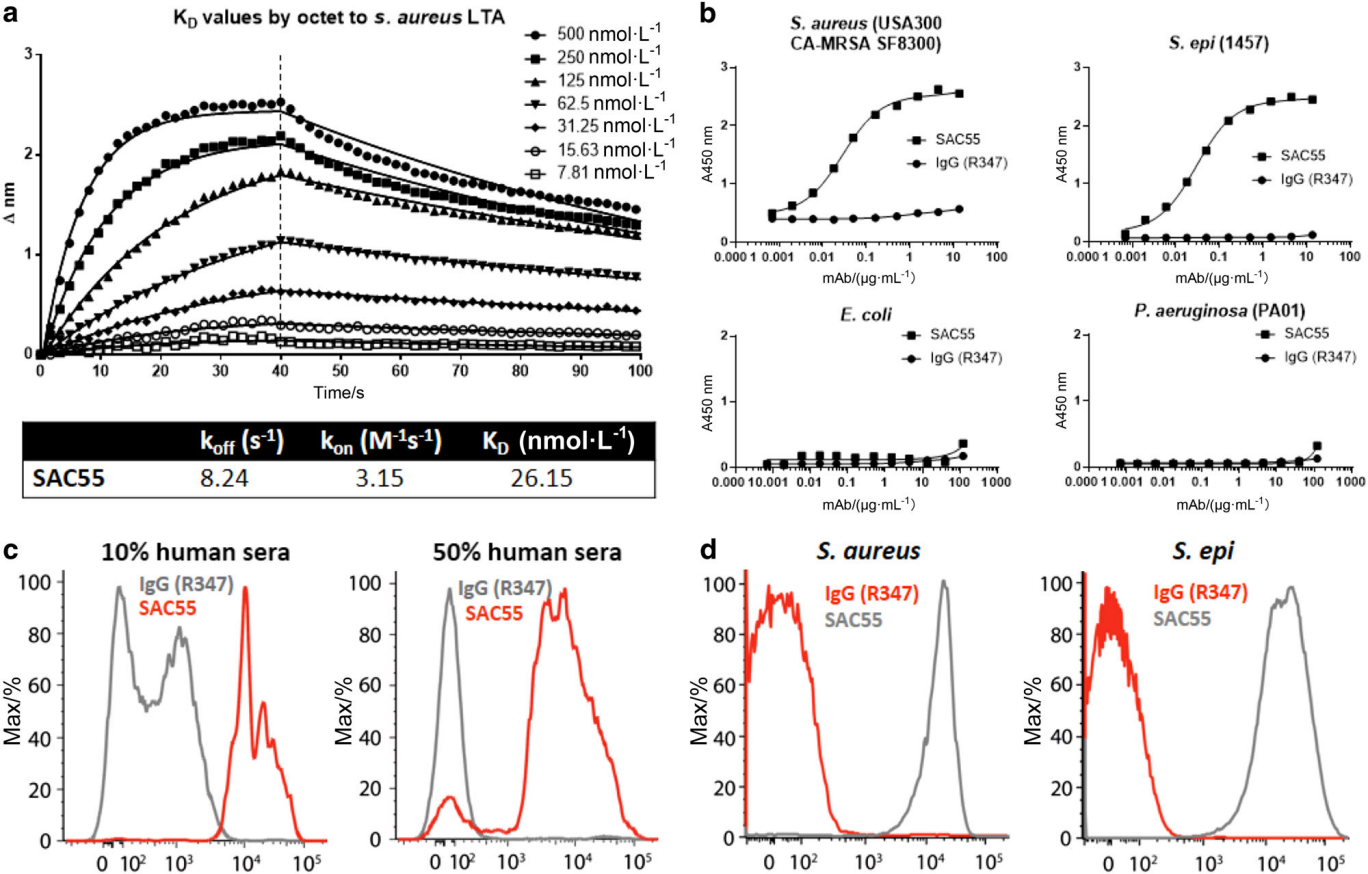
Anti-LTA mAb characterization. Experimental anti-LTA antibody (SAC55) was analyzed for binding characteristics by several methods against species-matched isotype control IgG (R347). **a** *k*_off_, *k*_on_, and *K*_D_ were assessed by Octet for SAC55 against LTA. **b** Selective binding of SAC55 to Gram-positive bacteria (*S*. *aureus* and *S*. *epidermidis*) but not Gram-negative bacteria (*E*. *coli* and *P*. *aeruginosa*) is assessed, as well as an absence of binding by the control antibody R347. **c** Flow cytometry of in vitro grown *S*. *aureus* reveals selective binding, with the distinction between groups improved by addition of human sera. **d** Flow cytometry of *S*. *aureus* harvested from mouse blood shows selective binding of SAC55 to Gram-positive bacteria, following in vivo passage.

In order to specifically measure the infectious entity itself, we developed an immunoPET agent against LTA, a cell-surface component of Gram-positive bacteria. An anti-LTA mAb (SAC55) was identified from memory B cells isolated from a patient recovering from an *S*. *aureus* skin infection. This human immunoglobulin G1 (IgG1) was evaluated for binding specificity and selectivity for LTA-positive strains of pathogenic bacteria with clinical significance. Affinity measurements by Octet of SAC55 binding to immobilized LTA showed strong binding for the target (Fig. 5a). SAC55 was also found by whole-cell enzyme-linked immunosorbent assay (ELISA) to bind specifically to LTA-positive bacteria, including *S*. *aureus* and *S*. *epidermidis*, but not to Gram-negative bacteria (specifically *Escherichia coli* and *Pseudomonas* *aeruginosa*), whereas the control IgG did not bind to any of the LTA-negative bacterial species tested (Fig. 5b)

SAC55 also bound to in vitro grown *S*. *aureus* and *S*. *epidermidis* as measured by flow cytometry in the presence of human sera (Fig. 5c), thus indicating that the binding epitope for this anti-LTA mAb is not obscured by the presence of human sera. Similarly, this anti-LTA mAb retained binding to the bacteria ex vivo, indicating that LTA is expressed, and its epitope is accessible for antibody binding on bacteria harvested from infected mice (Fig. 5d). These results indicate that SAC55 is a valid candidate to specifically detect LTA-expressing bacteria in an infected animal.

### Radiolabeling and immunoPET imaging of SAC55

**Fig. 6:**
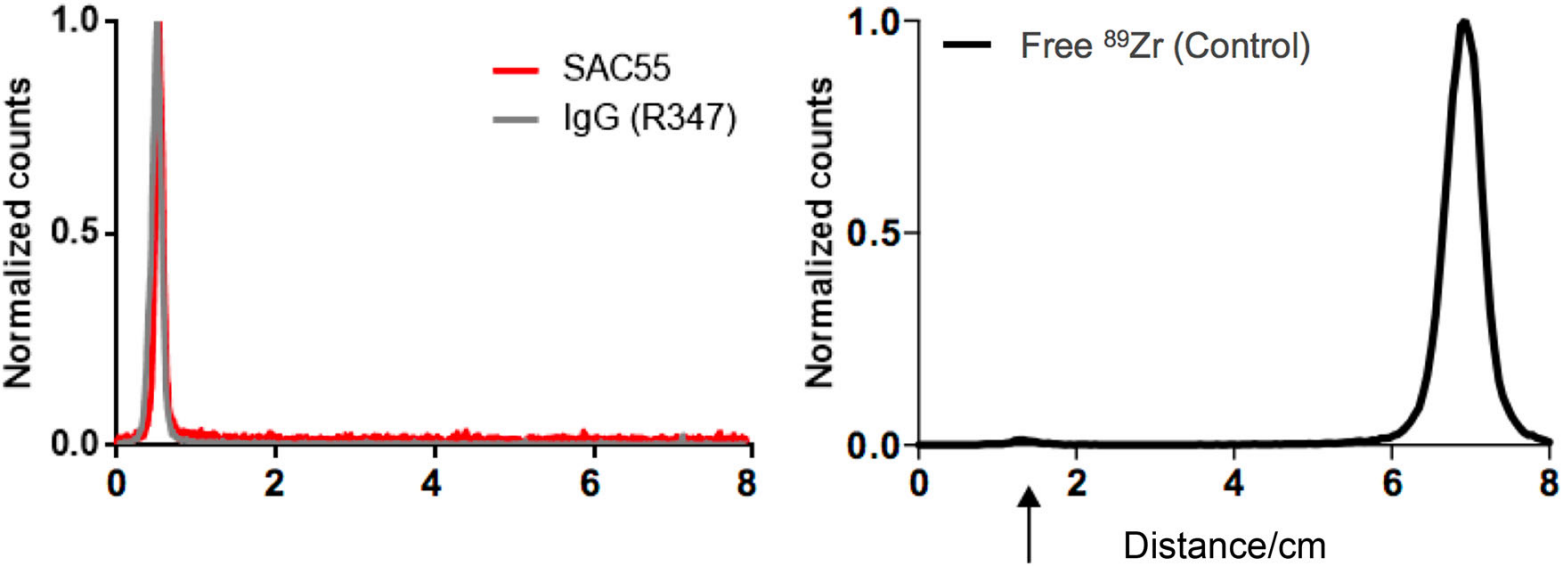
Radiopurity. Radiopurity of the immunoPET conjugates were determined by radio-instant thin layer chromatography.After purification, the activity for both SAC55 and R347 conjugates is retained at the origin, with no evidence of free zirconium migrating up the plate, indicating that injected material does not contain free zirconium-89.

Anti-LTA and isotype control IgG were covalently conjugated to deferoxamine (DFO) via an isothiocyanate-amine reaction covalent linkage and subsequently radiolabeled with zirconium-89. Post-purification radioTLC confirmed purity by showing the >98% radiopurity of the labeled constructs (Fig. 6).

**Fig. 7:**
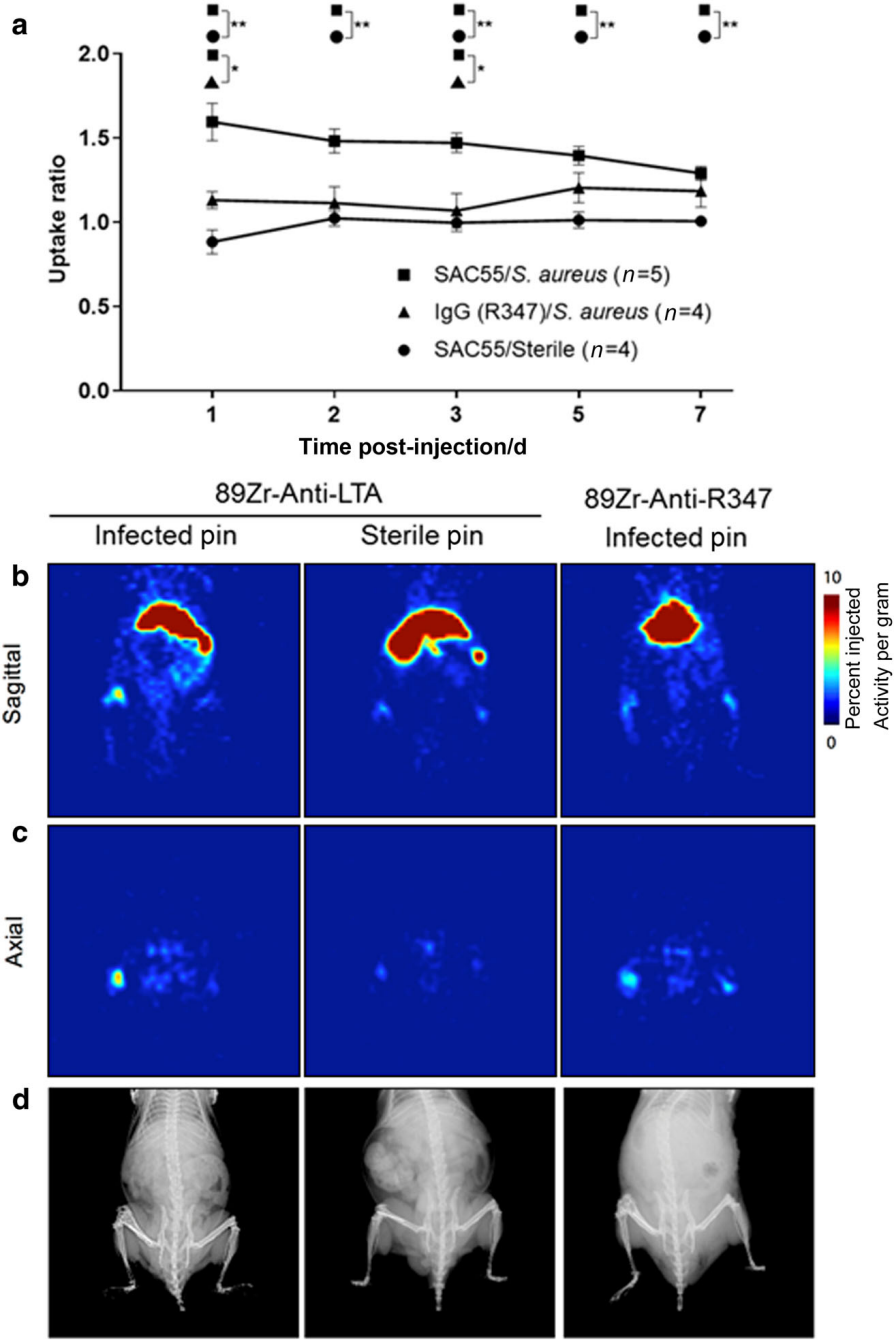
Uptake of ^89^Zr-labeled anti-LTA and control antibodies in vivo. Anti-LTA (SAC55) and control IgG mAbs were conjugated to DFO, radiolabeled with ^89^Zr, and injected into mice with (infected) and without (sterile) *S*. *aureus* inoculation in the in vivo model of PJI. **a** The ratio of postoperative leg signal to contralateral, non-operative leg signal is plotted as mean ± S.E.M. **b**, **c** Representative PET scans at day 1 after injection of [^89^Zr]SAC55 or [^89^Zr]IgG mAb depicted sagitally (**b**) and axially (**c**). **d** Representative X-ray images of the same mice, depicting implant location and morphological bone changes. *P < 0.05, **P < 0.01.

ImmunoPET imaging of infected implant mice with [^89^Zr]SAC55 and control [^89^Zr]IgG, as well as control sterile implant mice was conducted over 168 h. On day 1 post injection, animals receiving the anti-LTA antibody had a greater signal uptake ratio than infected implant mice receiving control IgG or sterile implant mice receiving the anti-LTA antibody (Fig. 7a). This was a surprising finding as the pharmacokinetics of full IgG, which may circulate for days or weeks, are such that the antibody typically accumulates over days as background signal reduces. As shown in the representative animals in Fig. 7b, early time points enabled clear distinction of the infected site using the specific antibody over the non-specific control or in the sterile infection model (*P* < 0.05 up to day 3). The specific contrast decreased over the 7 days of the experiment as non-specific zirconium-89 uptake in the skeleton increased.

A correlation analysis between of the uptake ratio of [^89^Zr]-labeled antibody and [^18^F]FDG and [^18^F]NaF was performed, in addition to the SUVmean values of [^89^Zr]-labeled antibody and BLI in the leg that had undergone surgical implantation of the pin. There was no significant correlation between the different PET imaging tracers [with *R*^2^ values (and two-tailed *P*-values) of 0.292 2 (0.069 6) and 0.137 4 (0.212 5), respectively]. A weak but statistically significant correlation between the standardized uptake values (SUVmean) and the bioluminescent signal yielded an *R*^2^ value of 0.588 8 (*P* = 0.003 6). These correlation results indicate specificity of the antibody-targeted approach with bacterial luminescence, while there is a lack of direct association between radiolabeled probes. While further work is required, this data indicate that a bacterial-specific tracer such as [^89^Zr]SAC55 may have considerable value in evaluating preclinical models and could assist in detection of PJI in the clinical setting.

## Discussion

Diagnosis of PJI in the setting of background inflammation is complex. Commonly used X-ray, CT, and MRI may not reliably distinguish sterile joint loosening from infection. Development of a bacteria-specific imaging probe would allow for differentiation of infectious processes from sterile inflammation. The sensitivity of PET imaging would be particularly valuable in settings of low bacterial burden. Here we evaluated a human mAb directed against LTA, a component of the cell wall of Gram-positive bacteria (implicated in >60% of PJI).^24^ The high-affinity mAb, SAC55, exhibited specific binding to Gram-positive *S*. *aureus* and *S*. *epidermidis* not only after in vitro growth (Fig. 5b, c) but also after in vivo passage in mice (Fig. 5d). With the intent to specifically and noninvasively image the bacterial pathogen directly and to facilitate improved sensitivity of detection, we have developed [^89^Zr]SAC55. This immunoPET agent provided statistically significant distinction of both the presence of infection over sterile implant sites as well as targeted over non-specific antibody.

Typically, immunoPET imaging has been conducted several days after administration as background blood pool clearance of the antibody is required to distinguish the site of interest. Consequently, we utilized the relatively long-lived isotope zirconium-89. However, we observed that the signal ratio of [^89^Zr]anti-LTA mAb at the *S*. *aureus*-infected postoperative legs vs contralateral, non-operative legs was greatest at 1 day after administration. We speculate that this may be in part due to the improved immune system recognition of the bacteria after antibody binding, leading to the clearance of both the bacteria and the tracer from the site. Shortening the time between injection and imaging would improve the logistics of utilizing this technology in patients and may allow for the use of shorter-lived isotopes, resulting in reduced radiation exposure.

SAC55 was compared to conventional PET tracers and CT imaging. [^18^F]FDG has previously been explored as a non-specific probe for evaluating PJI with varying success.^18^ PET provides insight into the metabolism of host tissue, as cells that utilize radioglucose trap the tracer locally. PET provides millimeter-scale tomographic images of tracer distribution throughout the whole body, while CT provides higher-resolution attenuation maps of use for anatomical localization, abnormal tissue findings, and to identify sites of bone degeneration. We have shown that FDG uptake in the surgical distal femur of infected mice was much greater than that of the contralateral, nonsurgical femur and also correlated with the BLI findings in the surgical vs nonsurgical sites. However, we also found increased FDG uptake in the sterile surgical leg (Fig. 2b), suggesting that some uptake is due to inflammation caused by the surgery. While [^18^F]FDG may easily identify robust PJI, indolent infections with low bacterial burdens, or those being actively treated, may be indistinguishable from sterile background inflammation.^25^

[^18^F]NaF has previously been used to assess the impacts of tuberculosis and total hip arthroplasty, as it is taken up by the surrounding bone matrix at these degenerative sites.^19–21^ While gross morphological changes were seen in this study after months of infection in the mouse model, such changes may not be distinguishable in the absence of long-term infection. Remodeling of the bone due to sterile inflammation may again preclude the distinction of indolent infection vs sterile inflammation, as confirmed when comparing the sterile implant to the contralateral knee (Fig. 3b). While CT and [^18^F]NaF PET may not aid in early diagnosis of PJI, they may be useful once substantial remodeling has occurred. A correlation analysis across the PET imaging probes and that of bacterial-specific bioluminescence indicates the specificity of the antibody-targeted PET imaging approach.

This study was limited in part by the fact that the physes in mice are continually active and do not fuse, as occurs in humans. This results in sustained bone turnover and signal at the ends of the long bones by both [^18^F]FDG and [^18^F]NaF.^26^ However, this confound was controlled for by comparing postoperative joints to their contralateral, non-operative sites, which also have an active physis. There are also practical considerations that are relevant when comparing different imaging tracers or modalities of translational import. While it is finding greater acceptance in the cancer-imaging realm, [^89^Zr]-labeled antibody PET imaging is still performed at only a small number of sites worldwide. CT imaging, [^18^F]FDG, and [^18^F]NaF PET are routinely performed and can provide same day (outpatient) logistics. Often with mAbs, the long clearance time after injection requires several days between administration and imaging. Intriguingly, here we have observed that the optimal [^89^Zr]SAC55 imaging time point was only 24 h after injection. BLI of bacteria in small animals is generally feasible but requires genetic manipulation of the infectious organism that may alter properties of the model and is not suitable for larger animal models or human use (as the light-producing proteins are highly immunogenic and provide signal sufficient for superficial imaging).

In conclusion, we have demonstrated the ability of an anti-LTA mAb immunoPET imaging probe to discriminate infection from non-specific inflammation in a preclinical mouse model of PJI. We have further highlighted the potential and shortcomings of conventional PET tracers in imaging of these models. We plan to examine the role of molecularly specific imaging to monitor efficacy of the SAC55 mAb as a targeted therapeutic to clear LTA-positive infections. Investigation in larger animal models and in patients is needed to determine whether the [^89^Zr]SAC55 probe can be translated to diagnose PJI and other infections in clinical practice.

## Materials and methods

### Antibody development

The monoclononal antibody (mAb) specific for LTA (anti-LTA, SAC55) was identified from IgG^+^ memory B cells selected from a patient convalescing from a *S*. *aureus* skin infection (erysipelas), as previously described.^27^ A human IgG1 mAb (R347, directed against an unrelated HIV-gp120 antigen) was used as an isotype control human IgG1.^28^

### Antibody labeling

The heterobifunctional chelate, p-SCN-Bn-DFO, was from Macrocyclics (Dallas, TX). Reagents and chemicals were from Sigma-Aldrich (St. Louis, MO) unless otherwise stated. Zirconium-89 was from the Mallinckrodt Institute of Radiology (Washington University Medical School, St. Louis, MO).

### Conjugating the chelator (DFO) to the antibody

To antibody in 0.1 mol · L^−1^ HEPES at pH 8.5, 10 µL of DFO in dimtethyl sulfoxide (233.3 µmol · L^−1^) was added three times followed by mixing to a final DFO:antibody ratio of 7:1. The reaction was mixed at room tempterature for 30–60 min. Excess unreacted DFO was removed by centrifugation using Amicon Ultra 0.5 mL Centrifugal Filters Ultracel 50 K Regenerated Cellulose 50000 NMWL (EMD Millipore, Billerica, MA).

### Labeling with ^89^Zr

To zirconium-89, an excess of 1 M oxalic acid was added. Slowly Na_2_CO_3_ was added to bring the pH to 7–7.5. ^89^Zr was added to the DFO-conjugate antibody, and the reaction was mixed at room temperature for 40 min. To chelate free ^89^Zr, 50 mmol · L^−1^ EDTA, pH 5, was used and removed by centrifugation using sterile saline in Amicon Ultra 0.5 mL Centrifugal Filters Ultracel 50 K Regenerated Cellulose 50000 NMWL (EMD Millipore, Billerica, MA). Instant thin layer chromatography (ITLC) was performed using silica impregnated filter paper (Pall Corporation, Port Washington, NY). The ITLC was run in 50 mmol · L^−1^ EDTA, pH 5, and subsequently imaged and quantified using the Phosphorimager and the AutoQuant software package, respectively (Packard, PerkinElmer, Hopkinton, MA). For both specific SAC55 and control antibodies, specific activities were in the range of 1.7–2.2 mCi · g^−1^.

### Bacteria and inoculum preparation

Community-acquired methicillin-resistant *Staphylococcus aureus* (CA-MRSA) strain SF8300 USA300 CA-MRSA was kindly provided by Binh Diep of UCSF (San Francisco, CA). *Staphylococcus epidermidis* strain 1457, *P. aeruginosa* strain PAO1, and *Escherichia coli* strains were obtained from American Type Culture Collection (ATCC, Manassas, VA).* S*. *aureus* and *S*. *epidermidis* were streaked for isolation on trypticase soy agar (TSA) plates (VWR International, Radnor, PA) and a single colony was inoculated into tryptic soy broth (TSB) and grown overnight. *P. aeruginosa* was grown overnight on a TSA plate and resuspended in TSB to the appropriate optical density (OD)_600 nm_. A bioluminescent CA-MRSA strain (SAP231) was derived from the parent NRS384 strain as previously described.^13^ SAP231 possesses the bioluminescent *lux* construct in the bacterial chromosome so that only live and actively metabolizing bacteria will produce light, and light production is maintained in all progeny without selection. Mid-logarithmic phase bacteria were obtained after overnight culture in TSB at 37 °C with shaking (240 r · min^−1^) followed by a 2 h subculture at 1:50 dilution. The desired inoculum of bacteria was washed and reconstituted in sterile phosphate-buffered saline (PBS).

### Whole-cell ELISA binding

Bacteria from an overnight culture were washed in ice-cold PBS (VWR International) and adjusted to an OD_600 nm_ ~ 0.1 in ice-cold PBS. ELISA plates (Nunc MaxiSorp, Nalge Nunc International, Rochester, NY) were coated with 100 µL of bacteria and incubated overnight at 4 °C. Plates with *S*. *aureus* were then blocked with 400 µg · mL^−1^ rabbit IgG (Sigma-Aldrich, St. Louis, MO), and plates with other bacterial species were blocked with 1% bovine serum albumin (BSA; Sigma-Aldrich, St. Louis, MO) in PBS for 2 h at 4 °C and then washed 3× with 0.1% Tween-20 (vol/vol) in PBS (PBST). Serial dilutions of 100 µL of SAC55 or isotype control R347 were then applied to the plates and incubated for 2 h at 4 °C. The plates were washed 3 times with PBST, and bound antibody was detected with 100 µL of 1 µg · mL^−1^ horseradish peroxidase-conjugated goat-anti-human IgG (Jackson Immunoresearch Laboratory, West Grove, PA) and 3,3′,5,5′-tetramethylbenzidine substrate (KPL, SeraCare, Milford, MA). The reaction was stopped after 10 min with 100 μL 0.2 mol · L^−1^ H_2_SO_4_, and the OD_450_ was measured in a spectrophotometer (Molecular Devices, Sunnyvale, CA).

### Flow cytometry—in vitro grown bacteria

Flow cytometry on in vitro grown bacteria was conducted on overnight bacterial cultures washed in ice-cold PBS, adjusted to OD_600 nm_ ~ 0.2, and blocked with 10% or 50% human sera for 1 h at 4 °C. After one wash in PBS with 0.1% BSA, pellet was incubated with 100 µL of Alexa Fluor 488 (ThermoFisher Scientific, Waltham, MA)-labeled SAC55or R347 at 10 µg · mL^−1^ in PBS in the presence of 10% or 50% human sera. Following two washes, mAb binding was quantified by flow cytometry with an LSRII flow cytometer (Becton-Dickinson, Franklin Lakes, NJ).

### Flow cytometry—in vivo grown bacteria

Six-to-eight-week-old female CD1 mice (Harlan, Indianapolis, IN) were infected by intraperitoneal injection of 5 × 10^8^ CFU of *S*. *aureus* or *S*. *epidermidis*. Four hours postinfection, the animals were euthanized, and blood obtained after cardiac puncture was pooled from 4 mice into ice-cold sodium citrate at 0.35% (weight:vol) final concentration. Eukaryotic cells were lysed with 1% NP40 (Thermo Fisher Scientific, Waltham, MA), and bacteria were recovered by centrifugation (10 min) at 8 000 r · min^−1^ at 4 °C. The bacterial pellet was sonicated in 2 mL ice-cold PBS, washed once in PBS, and transferred to a 96-well U-bottom plate (Thermo Fisher Scientific). First, non-specific binding to protein A was blocked with rabbit anti-protein A immune sera (1:100) for 30 min at 4 °C. Bacteria were then incubated with anti-LTA mAb or IgG1 (10 µg · mL^−1^) for 1 h at 4 °C, washed in PBS, and incubated with Alexa Fluor 633 (Thermo Fisher Scientific)-conjugated goat anti-human IgG for 30 min at 4 °C (Jackson Immunoresearch Laboratory, West Grove, PA). Following one wash, live bacteria were stained for 15 min at room temperature with BODIPY FL Vancomycin and mAb binding was quantified as above.

### Octet affinity measurement

Anti-LTA mAb-binding kinetics to purified LTA were analyzed using an Octet 384 instrument with 384 slanted well plates (ForteBio, Menlo Park, CA). An aminopropylsilane biosensor was first loaded with 100 µg · mL^−1^ of purified *S*. *aureus* LTA (Sigma-Aldrich) for 300 s. Anti-LTA mAb (7.8–500 nmol · L^−1^) association was measured for 40 s, followed by a 300 s dissociation into kinetic buffer (ForteBio). All steps were performed using a 3-mm sensor offset with 0.6 Hz sensitivity. Data were exported to Prism (GraphPad, La Jolla, CA) for Global Association/Dissociation affinity curve fitting.

### Mouse model of PJI

All animal procedures were approved by the Johns Hopkins University Animal Care and Use Committee. Six-week-old male C57BL/6 mice (Jackson Laboratories, Bar Harbor, ME) were used in all experiments. A mouse model of PJI was performed as previously described.^14,15^ Briefly, a medial parapatellar approach was used to access the right distal femur. After dislocating the patella, a 25-gauge needle was used to ream the distal femoral medullary canal. Thereafter, a medical-grade Kirschner-wire (0.5 mm diameter × 9 mm in length; Modern Grinding, Port Washington, WI) was inserted in retrograde fashion with approximately 0.5 mm protruding into the knee joint to which was added 2 µL sterile saline for the sterile control or directly inoculated with 1 × 10^3^ CFU. The patella was relocated and the knee was surgically closed using two interrupted absorbable sutures.

### In vivo BLI

Postsurgical infection was confirmed by direct BLI of bacteria using the IVIS Lumina III imaging system (PerkinElmer, Hopkinton, MA). Imaging (large binning and a 5 min exposure) was performed immediately on anesthetized mice (2% isoflurane) before surgery and then on postoperative days 3, 7, 14, and 21. Signal was quantified using the Living Image software within a region of interest (ROI) of 0.5 × 0.75 cm^2^ centered over the surgical knee and measured as maximum radiance (photons/second/cm^2^/steradian). The limit of detection was 2.5 × 10^3^ photons/s/cm^2^/sr. After an infection was established, the right hind limb was cleared of hair, and a bioluminescent image was acquired (IVIS Spectrum; Perkin Elmer). An elliptical ROI of 0.74 × 0.98 cm was used to measure persistent infection.

### Radiotracer administration

All animal work was in accordance with AAALAC, IACUC, and was approved by the Johns Hopkins Animal Care and Use Committee (Protocol #: MO15M421 and MO14M57). Clinical-grade [^18^F]FDG and [^18^F]NaF were obtained from the Johns Hopkins PET Center Radiopharmacy, supplied by PETNET Solutions (Siemens Healthcare, Malvern, PA).^26^ Injectate was assayed using a sodium iodide CRC-15 dose calibrator and a calibration factor of 439 (Capintec, Ramsey, NJ). Mice were anesthetized by isoflurane (2%) inhalation and injected intravenously via the retro-orbital sinus with 200 μCi average activity of tracer diluted to a final volume of 100 μL in isotonic saline. For [^18^F]FDG, PET/CT was performed following a 1 h uptake period under continuous isoflurane anesthesia. For [^18^F]NaF, the mice were scanned following a 40 min uptake period without anesthesia.

Radiolabeled antibodies were prepared as detailed above, and injectate was assayed using a well counter with a calibration factor of 517, as determined previously.^29^ In a volume of 150 µL, 225 µCi of radiolabeled antibody (approximately 100 µg) was administered intravenously via the retro-orbital sinus. Mice were serially scanned (10–20 min acquisitions, 350–750 keV energy window) on days 1, 3, 5, and 7 on a microPET R4 system (Concorde Microsystems Inc., Knoxville, TN) followed by Faxitron MX-20-DC12 digital X-ray imaging system (Faxitron Bioptics, LLC, Tucson, AZ).

### PET/CT imaging

Sequential PET (12 min acquisition, 140–700 keV energy window) and CT scans were performed using the SuperArgus small-animal integrated PET/CT scanner (Sedecal Systems, Buffalo Grove, IL). CT parameters for acquisition were 40 kVp and 0.8 mA with 2 mm aluminum filtration. The 3D imaging analysis platform Amira (version 5.0; FEI, Hillsboro, OR) was used to merge the PET and CT data sets to visualize [^18^F]-FDG uptake in relation to the surgical site.

### PET and high-resolution X-ray imaging

The dedicated microPET R4 system was used to acquire PET scans for radiolabeled antibodies. List-mode data were acquired using a gamma-ray energy window of 350–750 keV and a coincidence timing window of 6 ns. PET image data were corrected for detector nonuniformity, dead time, random coincidences, and physical decay. For all static images, scan time was between 10 and 20 min. Data were sorted into 3D histograms by Fourier rebinning, and transverse images were reconstructed using a maximum a priori algorithm to a 128 × 128 × 63 (0.845 mm × 0.845 mm × 1.211 5 mm) matrix. Data sets were analyzed using the ASIPro VM microPET analysis software. Volumes of interest were manually defined around the distal femur, and the injected activity per gram was calculated. An empirically determined system calibration factor for mice was used to convert voxel count rates to activity concentrations (in µCi per mL of tissue). Figures were generated using Amira (version 5.0; FEI, Hillsboro, OR). Directly after PET scanning, while in the same position, planar X-ray images of the mice were acquired using the Faxitron MX-20-DC12 digital X-ray imaging system (Faxitron Bioptics, LLC, Tucson, AZ).

### Statistical analysis

All data with statistics are expressed as mean ± S.E.M. Data were analyzed using an unpaired one-tailed (BLI and [^18^F]FDG and [^18^F]NaF uptake) or two-tailed (CT volume) Student's *t*-test with Welch’s correction or the Holm–Sidak method, with alpha = 0.05, and each row was analyzed individually, without assuming a consistent standard deviation. Analysis of variance followed by Bonferroni’s multiple comparison test was used to assess [^89^Zr]-antibody uptake; outliers were removed via analysis of absolute variance relative to the mean. Pearson’s correlation analysis was performed in Prism (GraphPad version 7.0d) between PET imaging of [^89^Zr]-antibody (using the ratio of uptake in the surgical vs contralateral leg and fold increase uptake of [^18^F]FDG and [^18^F]NaF) and the standardized uptake value (SUVmean) of [^89^Zr]-antibody in the surgical pin leg vs bioluminescent flux values from the same limb. *P*-values <0.05 were considered significant.

## Electronic supplementary material

Supplemental Figure 1. Correlation analysis between PET imaging tracers, and antibody and BLI

Supplemental Figure legend
