# Supplementary figures and images for: Molecularly specific detection of bacterial lipoteichoic acid for diagnosis of prosthetic joint infection of the bone
Source: Bone Res. 2018 Apr 25;6:13. doi: 10.1038/s41413-018-0014-y (PMC5916877; doi:10.1038/s41413-018-0014-y)

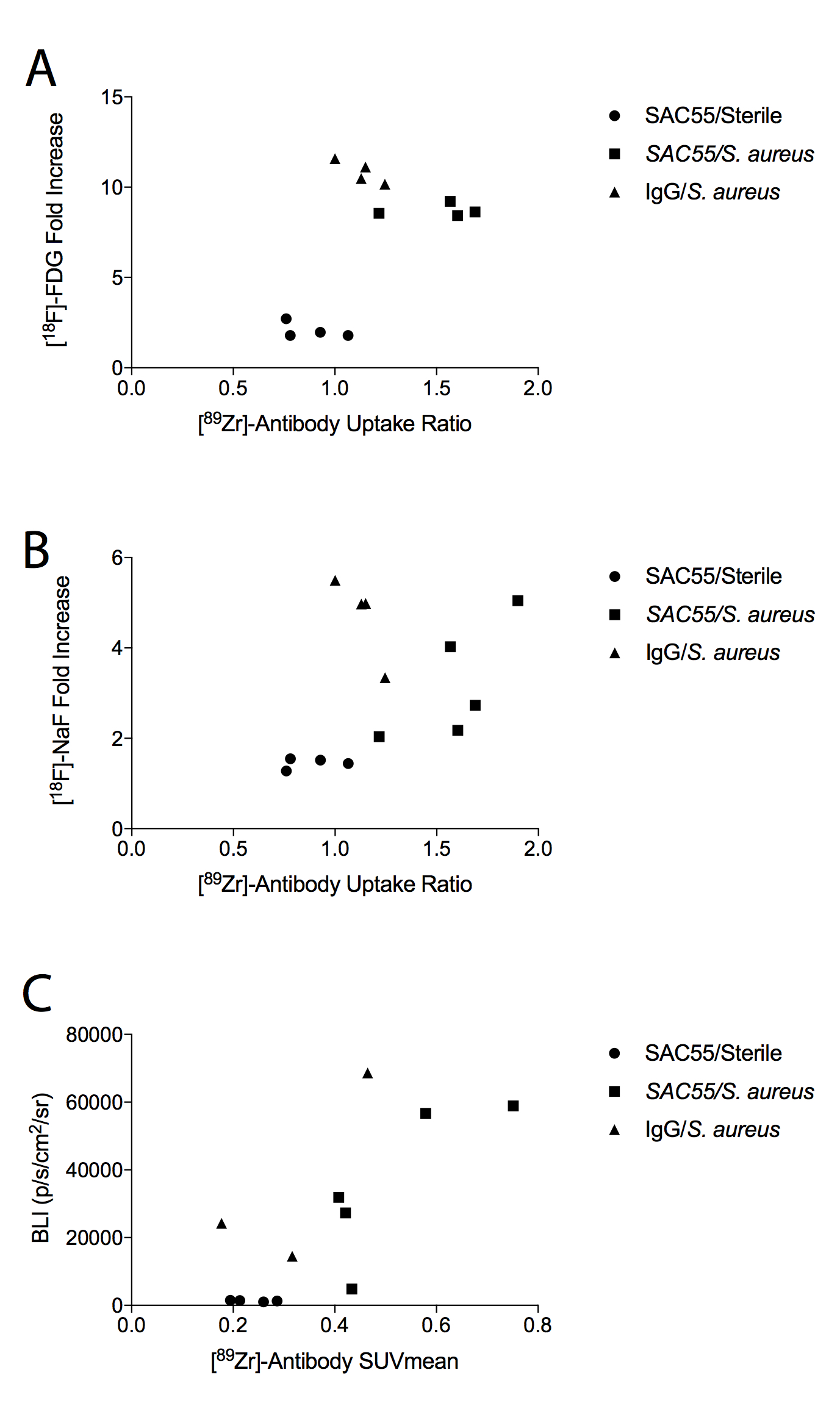

Supplement: Supplementary file 1 — Supplemental Figure 1. Correlation analysis between PET imaging tracers, and antibody and BLI [file 41413_2018_14_MOESM1_ESM.jpg]
