# Supplementary material for: Molecularly specific detection of bacterial lipoteichoic acid for diagnosis of prosthetic joint infection of the bone
Source: Bone Res. 2018 Apr 25;6:13. doi: 10.1038/s41413-018-0014-y (PMC5916877; doi:10.1038/s41413-018-0014-y)
Supplement: Supplementary file 2 — Supplemental Figure legend [file 41413_2018_14_MOESM2_ESM.docx]

Supplemental Figure 1. Correlation Analysis Between PET Imaging Tracers, and Antibody and BLI.

A) The ratio of [^89^Zr]-labeled antibodies uptake between the implant bearing and contralateral leg plotted against the fold-increase in [^18^F]FDG signal, or B) [^18^F]NaF. C) The SUVmean values of the [^89^Zr]-labeled antibody uptake in the surgically implanted knee against the maximum radiance in photons/cm^2^/steradian/second. Groups of infected or sterile-implanted animals receiving [^89^Zr]SAC55 or [^89^Zr]IgG, denoted by legend.
